# Continuous monitoring of brain dynamics with functional near infrared spectroscopy as a tool for neuroergonomic research: empirical examples and a technological development

**DOI:** 10.3389/fnhum.2013.00871

**Published:** 2013-12-18

**Authors:** Hasan Ayaz, Banu Onaral, Kurtulus Izzetoglu, Patricia A. Shewokis, Ryan McKendrick, Raja Parasuraman

**Affiliations:** ^1^School of Biomedical Engineering, Science and Health Systems, Drexel UniversityPhiladelphia, PA, USA; ^2^Nutrition Sciences Department, College of Nursing and Health Professions, Drexel UniversityPhiladelphia, PA, USA; ^3^Center of Excellence in Neuroergonomics, Technology, and Cognition, George Mason UniversityFairfax, VA, USA

**Keywords:** fNIRS, optical brain monitoring, working memory training, prefrontal cortex, hemodynamic response, wireless NIRS

## Abstract

Functional near infrared spectroscopy (fNIRS) is a non-invasive, safe, and portable optical neuroimaging method that can be used to assess brain dynamics during skill acquisition and performance of complex work and everyday tasks. In this paper we describe neuroergonomic studies that illustrate the use of fNIRS in the examination of training-related brain dynamics and human performance assessment. We describe results of studies investigating cognitive workload in air traffic controllers, acquisition of dual verbal-spatial working memory skill, and development of expertise in piloting unmanned vehicles. These studies used conventional fNIRS devices in which the participants were tethered to the device while seated at a workstation. Consistent with the aims of mobile brain imaging (MoBI), we also describe a compact and battery-operated wireless fNIRS system that performs with similar accuracy as other established fNIRS devices. Our results indicate that both wired and wireless fNIRS systems allow for the examination of brain function in naturalistic settings, and thus are suitable for reliable human performance monitoring and training assessment.

## Introduction

Understanding the neural mechanisms that contribute to cognitive functions such as performing complex cognitive tasks, acquisition, development, and use of cognitive skills, is an important goal for cognitive neuroscience research (e.g., Poldrack et al., 1998) and for applications of neuroscience to work and everyday activities, or neuroergonomics (Parasuraman, 2011). Various magnetic resonance imaging (MRI) methods have provided essential information about the brain systems involved in skill acquisition. These include functional MRI (Karni et al., 1995), resting state functional connectivity (Lewis et al., 2009), and diffusion tensor imaging (Lövdén et al., 2010). Such studies are critical for the development of theories of neuroplasticity, because human brain changes associated with learning can be compared to studies in animals using invasive neurophysiological and pharmacological methods (Sarter and Parikh, 2005). However, MRI has two major limitations; (1) its requirement for participant immobility, and (2) its high operational cost. The former rules out its use for understanding brain dynamics during everyday activities such as walking or running, and while many functional MRI studies have been carried out using virtual reality simulations of such naturalistic activities as spatial navigation (Hartley et al., 2003), flying (Callan et al., 2012), and driving (Calhoun et al., 2002), the concern is that carrying out these activities while prone and immobile may not recruit the same brain networks as those involved when one is mobile and upright (Raz et al., 2005). One consequence of the second limitation of MRI, its high operational cost, is that skill acquisition studies typically image participants pre- and post-training, so that only linear changes in brain structure and function can be assessed. However, skill acquisition is known to be non-linear, e.g., described by power or hyperbolic functions (e.g., Newell and Rosenbloom, 1980; Speelman and Kirsner, 2005). Also, neural changes are likely to occur throughout training, so it is important to examine how such changes are linked to performance at multiple time points during learning, not just before and after training.

One method that is well-suited for such continuous monitoring of brain dynamics is functional near infrared spectroscopy (fNIRS). fNIRS is a non-invasive, safe, and portable optical method to monitor the brain activity within the prefrontal cortex of the human brain. fNIRS has emerged during the last decade as a promising non-invasive neuroimaging tool and has been used to monitor various types of brain activities during motor and cognitive tasks with increasing interest from research communities. fNIRS uses specific wavelengths of light to provide measures of cerebral oxygenated and deoxygenated hemoglobin that are correlated with the fMRI BOLD signal (Cui et al., 2011). While fMRI can monitor the whole brain with high spatial resolution at the sub-millimeter level, fNIRS can only monitor cortical regions with less spatial resolution (usually in the centimeter range). However, unlike fMRI, fNIRS is quiet (no operating sound), provides higher temporal resolution (faster sampling frequency), and participants are not restricted to a confined space or are not required to stay in a supine position motionless. Hence, fNIRS is an ideal candidate for monitoring cortical activity related changes not only in laboratory settings but also in more ecologically valid, everyday working and field conditions.

In this paper we provide results from a number of studies that illustrate the use of this approach to the examination of workload and training-related brain dynamics with human performance assessment. This paper has two major aims. The first is to show that fNIRS provides a sensitive and reliable index of brain activity in skill acquisition, task performance and during the development of expertise in complex tasks. Because occupations involving such tasks often require their human operators to be free to speak, move their eyes or heads, and otherwise be mobile, a second aim of this paper is to describe a mobile fNIRS system that can supplement “tethered” fNIRS in studies of human performance. We first describe the use of fNIRS in monitoring cognitive workload in air traffic controllers. We then describe a study of the acquisition of a dual-tasking skill, in which participants had to perform challenging verbal and spatial working memory tasks simultaneously, as well as a study examining development of expertise in piloting unmanned vehicles. Each of these studies used conventional fNIRS devices in which the participants were tethered to the device while seated at a workstation. Current fNIRS instruments require the participant to be connected to the sensor and the device via cables and/or fiber optic lines which imposes restrictions on the ambulatory nature of the experiment protocol and to the participant. Consistent with the aims of mobile brain imaging (MoBI) (Makeig et al., 2009; Gramann et al., 2011) and neuroergonomics (Parasuraman, 2011), it would be desirable to measure brain dynamics while participants can move freely. To address these limitations we introduce a compact and battery-operated wireless fNIRS system that performs with similar accuracy as other established fNIRS devices.

## Monitoring cognitive workload in air traffic controllers

The assessment of cognitive workload using neural measures is a central feature of research and development in neuroergonomics (Parasuraman, 2011). Mental workload is also a critical factor in maintaining safety in air traffic control (ATC), particularly as traffic density increases and new systems and operational procedures are implemented for air traffic management (Loft et al., 2007). Accordingly, there is a need for sensitive, objective methods of measuring cognitive workload in air traffic controllers.

**Figure 1:**
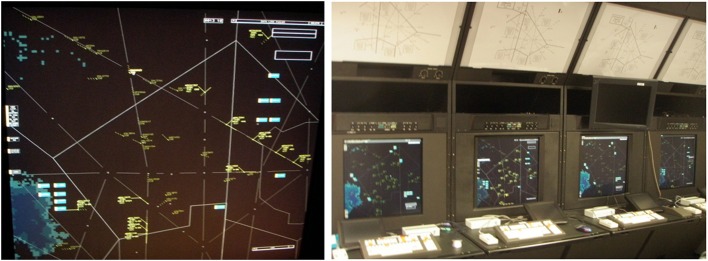
**Air Traffic Control simulator screenshot displaying a sector with en route aircrafts (left)**. Control workstations with high resolution radarscope, keyboard, trackball, and direct keypad access (**right**).

The feasibility of using fNIRS for human performance assessment has been demonstrated in several recent studies. In a prior research project, we incorporated fNIRS to studies conducted at the Federal Aviation Administration (FAA)'s William J. Hughes Technical Center Human Factors Laboratory where certified controllers were monitored with fNIRS while they managed realistic ATC scenarios under typical and emergent conditions (Ayaz et al., 2012b). The primary objective of this work was to use neurophysiological measures to assess cognitive workload and usability of new interfaces developed for ATC systems (see Figure 1). Throughout the study, certified professional controllers (CPCs) completed ATC tasks with different interface settings and controlled difficulty levels for verification. The results provide evidence that brain activation as measured by fNIRS provides a valid measure of mental workload in this realistic ATC task (Ayaz et al., 2012b).

For the first part of the study, we used a working memory task (N-back) that has been widely used in the cognitive neuroscience research literature (Owen et al., 2005). The N-back paradigm provides varying task-load conditions to test associations between level of difficulty and cortical activation, and has been shown to activate the dorsolateral (DLPFC) and ventrolateral prefrontal cortex (VLPFC) as assessed with Positron Emission tomography (PET) (Smith et al., 1996) and functional MRI (D'Esposito et al., 1998; Owen et al., 2005). During the task, participants were asked to monitor stimuli (single letters) presented on a screen serially and click a response button when a target stimulus arrives. Four conditions were used to incrementally vary working memory load from zero to three items. In the 0-back condition, subjects respond to a single pre-specified target letter (e.g., “X”) with their dominant hand (pressing a button to identify the stimulus). In the 1-back condition, the target is defined as any letter identical to the one immediately preceding it (i.e., one trial back). In the 2- and 3-back conditions, the targets were defined as any letter that was identical to the one presented two or three trials back, respectively. The total test included seven sessions of each of the four n-back conditions (hence, a total of 28 N-back blocks each of 1 min duration, which had 20 letters presented each for 500 ms with a 2500 ms inter-stimulus time) presented in a pseudo-random order.

**Figure 2:**
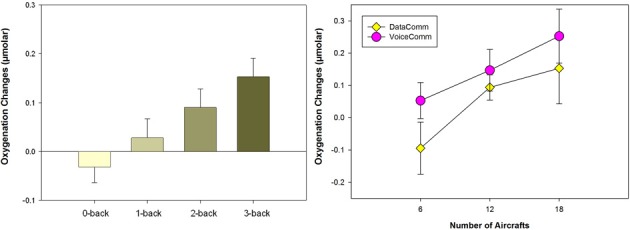
**(Left) Average oxygenation changes of all subjects (24 participants, and 28 trials for each participant) with increasing task difficulty**. (**Right**) Average oxygenation changes for two different interfaces (data- and voice-based) and for 6, 12, and 18 aircraft conditions each. Error bars are standard error of the mean (s.e.m.).

Results showed that average oxygenation changes due to task engagement (mean for each block with baseline compared to beginning of the block) at optode 2 that is close to AF7 in the International 10–20 System, located within left inferior frontal gyrus in the dorsolateral prefrontal cortex (DLPFC), were associated with task difficulty and increased monotonically with increasing task difficulty (see Figure 2, left). Moreover, the significant region within left PFC in this study was implicated in many previous studies of the N-back task using PET (Smith et al., 1996; Reuter-Lorenz et al., 2000), fMRI (Cohen et al., 1997; Owen et al., 2005), and fNIRS (Schreppel et al., 2008).

For the second part of the study, complex cognitive tasks (i.e., ATC) were used. A critical transition defined in the planned future ATC system called NextGen involves augmenting the current auditory-based communications between ATC and the flight deck with text-based messaging, or DataComm systems (Willems et al., 2006, 2010). DataComm systems are expected to allow controllers to manage more air traffic at a lower level of cognitive load, thereby increasing both the capacity of the national airspace system and passenger safety.

Based on the approach and findings of the N-back working memory study, the objective in the second part of the study was to use neurophysiological measures to predict changes in cognitive workload during a complex cognitive task that very closely simulated the activities of air traffic controllers. Two types of communications between the CPCs and pilots, either typical (VoiceComm) or emergent (DataComm) communications were used in ATC simulations in a pseudo-random order (Willems et al., 2006, 2010). For each communication type, task difficulty was varied by the number of aircraft in each sector, containing 6, 12, or 18 aircraft to increase task load. Three simulation pilots supported each sector within voice-based scenarios and entered data at their workstations to maneuver aircraft, all based on controller clearances.

Analysis of data from 24 participants indicated a significant measurement location of optode 8 which is within the medial PFC/frontopolar cortex, and there were two significant main effects, Task Difficulty (number of aircrafts) and Communication (VoiceComm vs. DataComm) (see Figure 2, right). The fNIRS results from the main effect of Communication type confirms that VoiceComm condition results in higher oxygenation compared to the DataComm condition with a small to moderate effect size. These results are consistent with the idea that, given the same cognitive workload (within identical scenarios), DataComm required fewer cognitive resources.

## Brain dynamics during extended working memory training

The previously described study by (Ayaz et al., 2012b) showed that frontal cerebral oxygenation as measured by fNIRS increases with working memory load. Specifically, average oxygenation systematically increased in the N-back task as N was varied from 0 to 3 (see Figure 1, left). Working memory capacity is predictive of performance on visual attention (Engle, 2002), decision-making (Endsley, 1995), and supervisory control tasks (McKendrick et al., 2013b). Hence it is of interest that recent studies have shown that an individual's working memory capacity is not fixed but can be increased by training (Jaeggi et al., 2008), although whether such training transfers to other general domains of cognition is controversial (Shipstead et al., 2012). MRI studies have also shown that such working memory training is associated with both structural (Takeuchi et al., 2010) and functional (Dahlin et al., 2008) brain changes. However, as mentioned previously, most studies have used MRI in pre- and post-training designs, so that fine-grained and non-linear changes in brain dynamics have not been studied. Accordingly, we describe a study that used fNIRS to monitor skill acquisition in a dual verbal and spatial working memory task (McKendrick et al., 2013a).

As people perform a task repeatedly, they are likely to experience changes in the degree of mental effort expended, either voluntarily or as required by the task. To distinguish between brain changes as a result of working memory training from increases in mental effort, we compared two training conditions in this study: An adaptive training condition in which working memory load was adjusted based on the trainee's performance, and a yoked condition whose working memory load was adjusted based on the performance of individuals in the adaptive condition. Since task demands are not matched to the capabilities of participants in the yoked group, we predicted that they would expend more mental effort in order to perform the task and show an increase in prefrontal cortex (PFC) total hemoglobin (HbT) as measured with fNIRS. At the same time, because task demands are matched to the capabilities of adaptive-trainees, we therefore expected this group to show little change in hemodynamic response in PFC.

**Figure 3:**
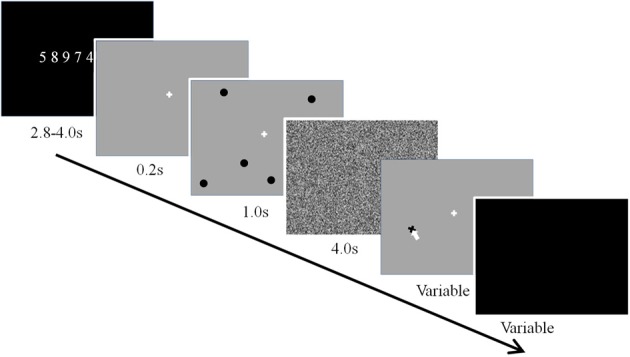
**Dual verbal-spatial working memory task**.

In addition, to improve the efficacy of the working memory training design, we implemented the suggestions for optimal training proposed by Gibson et al. (2012). First, we used a challenging dual verbal-spatial working memory task (see Figure 3), in which participants first memorized a string of digits, then a number of spatial locations, and following a delay period, recalled the locations and then the digits. The use of the dual task allowed for taxing the updating and executive control components associated with working memory (Baddeley, 1986). Second, to avoid ceiling effects and challenge participants, the load for verbal and spatial working memory was set to a range beyond what is considered the average capacity limit (spatial: 4 locations, verbal: 7 digits). Participants trained on the working memory task for about 2 h each day for 5 successive days. Daily training was separated into two 1 h sessions with a 15 min break between training sessions. Within a given training sessions participants performed 10 training blocks and each training block consisted of nine trials of the dual working memory task. Finally, we used linear mixed effects modeling of the data to examine both linear and non-linear changes in performance and brain dynamics with working memory training.

**Figure 4:**
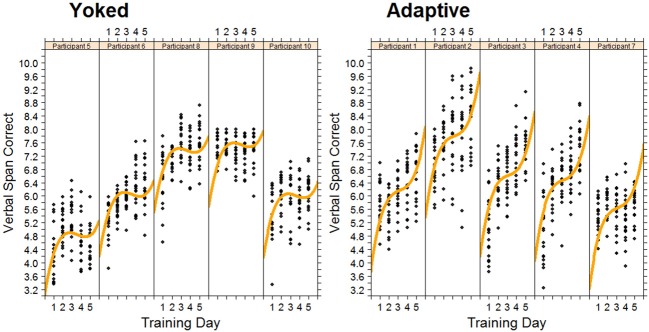
**Changes in Verbal span for each trainee in the adaptive training and yoked groups over 5 successive days of working memory training**. Each data point represents task performance during a training block.

Figure 4 shows the data, plotted for each individual, for the verbal working memory task. As is clear from Figure 4, verbal working memory span increased with training, but in a non-linear manner. A cubic function provided the best fitting model for training-related changes in performance. As expected, the adaptive group reached higher span levels than the yoked group at the end of training. Differences between training groups were modeled by a significant negative quadratic component for the yoked training condition, representing a slowing of skill development on the third and fourth days of training relative to the adaptive condition (see Figure 4).

**Figure 5:**
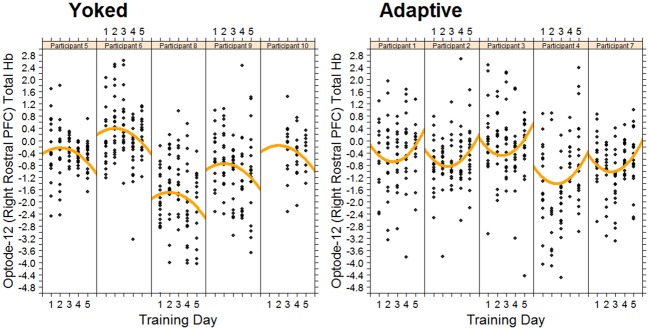
**Changes in total hemoglobin in right rostral PFC for each trainee in the adaptive training and yoked groups over 5 successive days of working memory training**. Each data point represents fNIRS measured brain activity during a block.

As predicted, we observed an increase in hemodynamic response for the yoked control condition. This was specifically observed in the right rostral prefrontal cortex during the first 3 days of training. In the same region, in the adaptive condition there was a decrease in hemodynamic response over the same time period. The rostral prefrontal cortex is believed to be involved in the monitoring and processing of sensory stimuli during multitasking (Burgess et al., 2005). The changes in rostral prefrontal cortex suggest that in order to keep pace with the performance of the adaptive group the yoked group had to apply considerably more effort in maintaining and processing dual task representations. This is expected as in the yoked group task demands are not matched with participant capabilities and thus require higher mental effort. Furthermore, toward the end of training the adaptive group had to increase the effort applied to processing dual task representations to improve their performance. The differential quadratic workload effect between adaptive and yoked conditions can be seen in Figure 5. During this time the yoked group may have become fatigued due to the high level of effort required on the first 3 days of training. Non-linear increases in left DLPFC and right VLPFC were also observed with increased exposure to working memory training.

These findings point to the sensitivity of fNIRS to track both linear and non-linear changes in cerebral hemodynamics as a result of working memory training. Importantly, non-linear changes over time would not have been observed if a pre-/post-training design commonly used in MRI studies had been used. The findings also show that fNIRS provides an efficient and effective way to continuously monitor hemodynamic changes over extended periods of time, as required in training studies. In addition, as described in this paper, portable NIRS systems are being developed that could be used to measure the effects of training in complex real world tasks where the use of fMRI would be challenging or impossible.

## Monitoring the development of expertise in piloting unmanned vehicles

The study by McKendrick et al. (2013a) described above used a basic but challenging cognitive task—dual verbal/spatial working memory. Expertise development in more complex tasks that simulate work and other everyday real settings has also been examined. A majority of the studies examining task practice have found decreases in the extent or intensity of neural activations with ongoing practice, particularly in the attentional control areas (Kelly and Garavan, 2005). This finding is true whether the task is primarily motor [e.g., golf swing (Milton et al., 2004)] or primarily cognitive in nature, as in the Tower of London task (Beauchamp et al., 2003) including more complex tasks such as videogame training (Prakash et al., 2012) or the center-out adaptation task (Gentili et al., 2013). Decreases in activation are thought to represent a contraction of the neural representation of the stimulus (Poldrack, 2000) or a more precise functional circuit (Garavan et al., 2000).

In a recent study, we utilized fNIRS to investigate the relationship of the hemodynamic response in the anterior prefrontal cortex to changes in the level of expertise, and task performance during learning of simulated unmanned aerial vehicle (UAV) piloting tasks (Ayaz et al., 2012a). Novice participants with no prior UAV piloting experience participated in a 9 day training program where they used a flight simulator to execute real world maneuvers. Each day, self-reported measures (with NASA TLX), behavioral measures (task performance), and fNIRS measures (prefrontal cortex activity as mental effort on task) were recorded.

Participants practiced approach and landing scenarios while piloting a virtual UAV. The scenarios were designed to expose novice subjects to realistic and critical tasks for a UAV ground operator directly piloting an aircraft. The first scenario was a turn-to-approach task, in which the pilot flies through several waypoints on an approach to land at an airfield. The second scenario was a landing task, in which the pilot performs the actual touchdown. In both scenarios, subjects were told to fly as smoothly as possible, learn the optimal paths, cope with crosswinds, and operate within certain speed and bank angle constraints. The experiment protocol involved a total of nine sessions per subject, one session per day. The first session on day 1 was to allow subjects to become acquainted with the flight simulator; by the end of this session, they needed to demonstrate basic understanding of flight simulator controls. Study data were collected during the following eight practice sessions.

**Figure 6:**
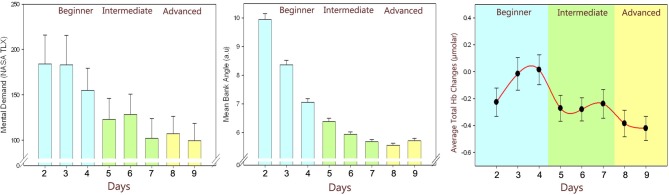
**Changes throughout the practice levels: Self-reported ratings: perceived mental effort as measured by NASA TLX (left), Behavioral performance: average error in banking angle (middle), fNIRS measures: average total hemoglobin concentration changes (right) of all subjects throughout days**. Error bars are standard error of the mean (SEM).

Analysis of data from thirteen participants showed a reduction in the fNIRS measures (see Figure 6), which were significantly different across practice levels and matches the same trends reported in behavioral performance and self-reported measures. A valid hypothesis can be derived from the evidence that expertise tends to be associated with overall lower brain activity relative to novices, particularly in prefrontal areas (Milton et al., 2004). Both practice and the development of expertise typically involve decreased activation across attentional and control areas, freeing these neural resources to attend to other incoming stimuli or task demands. As such, measuring activation in these attentional and control areas relative to task performance can provide an index of level of expertise and illustrate how task-specific practice influences the learning of tasks.

Results indicate that level of expertise does appear to influence the hemodynamic response in the dorsolateral/ventrolateral prefrontal cortices confirming previous studies with learning cognitive-motor tasks (Hatakenaka et al., 2007; Leff et al., 2008). Since fNIRS allows development of portable and wearable instruments, it has the potential to be used in future learning environments to personalize the training regimen and/or assess the effort of human operators in critical multitasking settings (Ayaz et al., 2012a, b).

## Development of a portable, wireless fNIRS system

The portable optical brain imaging system used in our studies reported here was first described by Chance et al. (Chance, 1998; Chance et al., 1993) further developed at Drexel University (Philadelphia, PA), manufactured and supplied by fNIR Devices LLC (Potomac, MD; www.fnirdevices.com).

**Figure 7:**
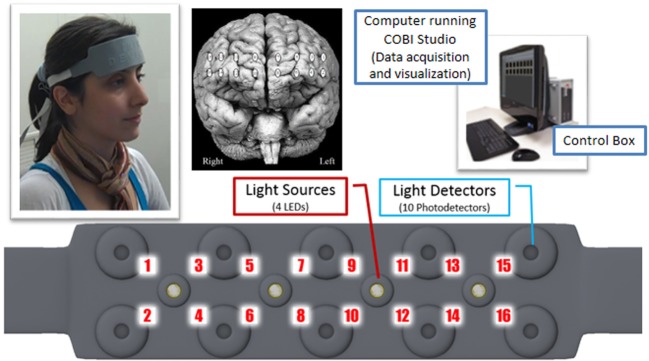
**Overview of fNIR system: Computer running COBI Studio (Drexel University) collects data through hardware control box**. Flexible Sensor housing 4 LED light sources, 10 photo-detectors provides 16 measurement locations.

The system is composed of three modules: a flexible headpiece (sensor pad), which holds light sources and detectors to enable a fast placement of all 16 optodes; a control box for hardware management; and a computer that runs the data acquisition (see Figure 7).

The sensor has a temporal resolution of 500 milliseconds per scan with 2.5 cm source-detector separation allowing for approximately 1.25 cm penetration depth and 16 measurement locations (optodes) on a rectangular grid covering the forehead region (see Figure 7) designed to monitor dorsal and inferior frontal cortical areas. The light emitting diodes (LEDs) were activated one light source at a time and the four surrounding photodetectors around the active source were sampled. For data acquisition and visualization, COBI Studio software was used (Ayaz et al., 2011).

### Evolution of Drexel wireless fNIRS

The need for development and improvement of fNIRS instrumentation has been growing as fNIRS has been increasingly used in human brain activation studies since it was first described by Jobsis (1977) as an optical method for non-invasively assessing cerebral oxygenation changes. In the 1980s, Delpy et al. designed and tested an fNIRS system for a clinical application with newborn infants (Wyatt et al., 1986). Further efforts improved the methodology and hardware (Delpy et al., 1988; Wray et al., 1988; Cope, 1991; Chance et al., 1993; Elwell et al., 1994) and thus expedited the translation of fNIRS based techniques into a useful neuroimaging tool (Villringer et al., 1993; Chance et al., 1997, 1998; Hoshi and Tamura, 1997; Villringer and Chance, 1997; Obrig et al., 2000; Strangman et al., 2002). Recent comprehensive reviews on fNIRS technology (Ferrari and Quaresima, 2012) and instrumentation (Scholkmann et al., 2013) confirm that the vast majority of instrumentation development was on continuous wave (CW) type fNIRS which is limited in terms of its information content (i.e., it measures only changes of oxygenated and deoxygenated-Hb) compared to frequency and time-resolved approaches. However, CW fNIRS is also most appropriate for miniaturization and portable system development, because the signal type and acquisition timing requirements are less demanding. Moreover, other than brain imaging, the same approach can also be used for many biomedical approaches (Macnab and Shadgan, 2012) such as muscle assessment and other *in vivo* and clinical applications.

The development of wearable and low cost fNIRS systems began in 1996 for prefrontal cortex brain hemodynamics and muscle measurements (Chance et al., 1997). These systems, were later further developed into the portable systems at Drexel University (Izzetoglu et al., 2004, 2005, 2011; Ayaz et al., 2012b) and used in the studies reported here.

One of the earliest wireless telemetry systems for fNIRS was based on a single channel muscle oximeter developed by Nakase and Shiga (Omron Institute of Life Science, Kyoto, Japan) in collaboration with Chance (Ferrari and Quaresima, 2012). This system was used by Hoshi et al. for assessing cognitive function of children that carried the system in their backpack, allowing them to move untethered (Hoshi and Chen, 2002). More recently, a 4-channel wireless *in vivo* imager has been developed at the University of Zurich, Switzerland (Muehlemann et al., 2008) and utilized for neurorehabilitation fNIRS studies (Holper et al., 2010). Also, an EEG integrated prototype was reported for epilepsy research (Safaie et al., 2013).

Consistent with the MoBI (Makeig et al., 2009; Gramann et al., 2011) and neuroergonomics (Parasuraman, 2011) approaches, development of miniaturized fNIRS systems for ubiquitous monitoring of the brain could benefit studies for understanding brain dynamics in ecologically valid real world environments.

### Two unit approach using smartphones

**Figure 8:**
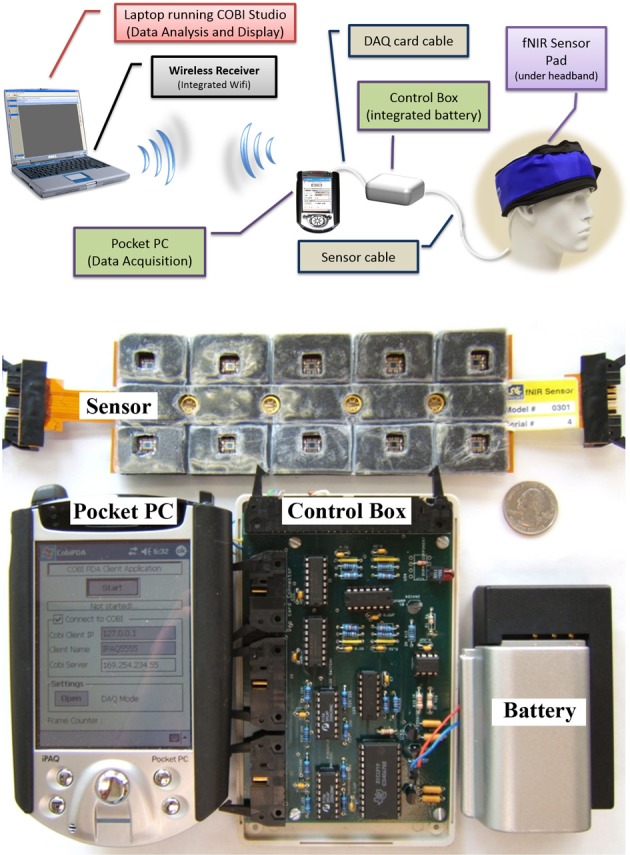
**General design concept (top) and implemented system components displaying 16-channel prefrontal cortex probe, pocket pc, control box, and battery (bottom)**. A quarter (US $0.25 piece) is also included for size comparison.

In this initial effort, our aim was to develop a miniaturized and battery operated CW fNIRS system with comparable operation features with the wired/portable system (Yurtsever et al., 2006), such as 16 channel full forehead assessment and 2 Hz sampling. To expedite the development and take advantage of the off-the-shelf embedded systems, a smartphone/pocket pc platform was utilized (Yurtsever et al., 2003). A custom software application and low level driver for direct hardware access was developed to interface a PCMCIA based National Instrument data acquisition card and control the optical imaging hardware unit and sampling of the data. The smartphone system simultaneously transmits the collected signals through a Wi-Fi network to a computer base station running COBI Studio software, thus allowing online monitoring at a remote base station (see Figure 8).

#### Control circuitry

The circuit board contains a stable current source for LEDs, implemented with a high precision voltage regulator, timing control elements (counter, demultiplexer, multiplexers), amplifiers, filters and is powered by a 7.2 volt Lithium-Ion camcorder battery. A 5 V voltage regulator is used to provide constant voltage to the circuit since battery voltage is decreased throughout operation.

The circuit is designed to use a minimum number of digital and analog channels of the data acquisition card, so that different data acquisition (DAQ) cards can be used with the control box. Only two digital channels and four analog channels are required to operate the system.

**Figure 9:**
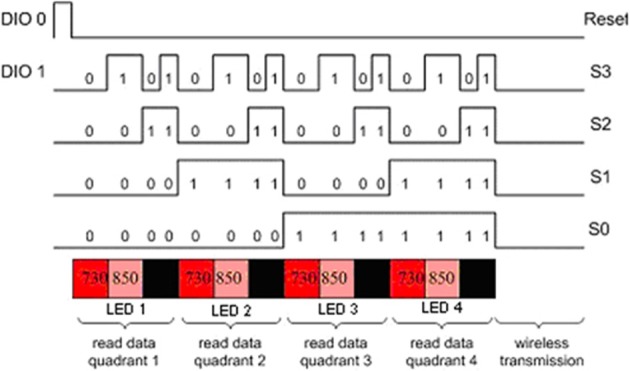
**Timing diagram of the control circuit**.

Controlling the timing of the LEDs and photodetectors is the key point in the design and were controlled by the data acquisition software in the Pocket PC. The LEDs turn on and off sequentially, one at a time. The LED turn on sequence in one scan cycle is depicted in red, pink and black colors below the timing signals in Figure 9. The LED turn on sequence is as follows: Turn on LED1 730 nm, read D1, D2, D3, D4 (Detectors 1–4); turn on LED1 850 nm, read D1, D2, D3, D4; dark, read D1, D2, D3, D4 (read offset); turn on LED2 730 nm, read D3, D4, D5, D6, and so on.

**Figure 10:**
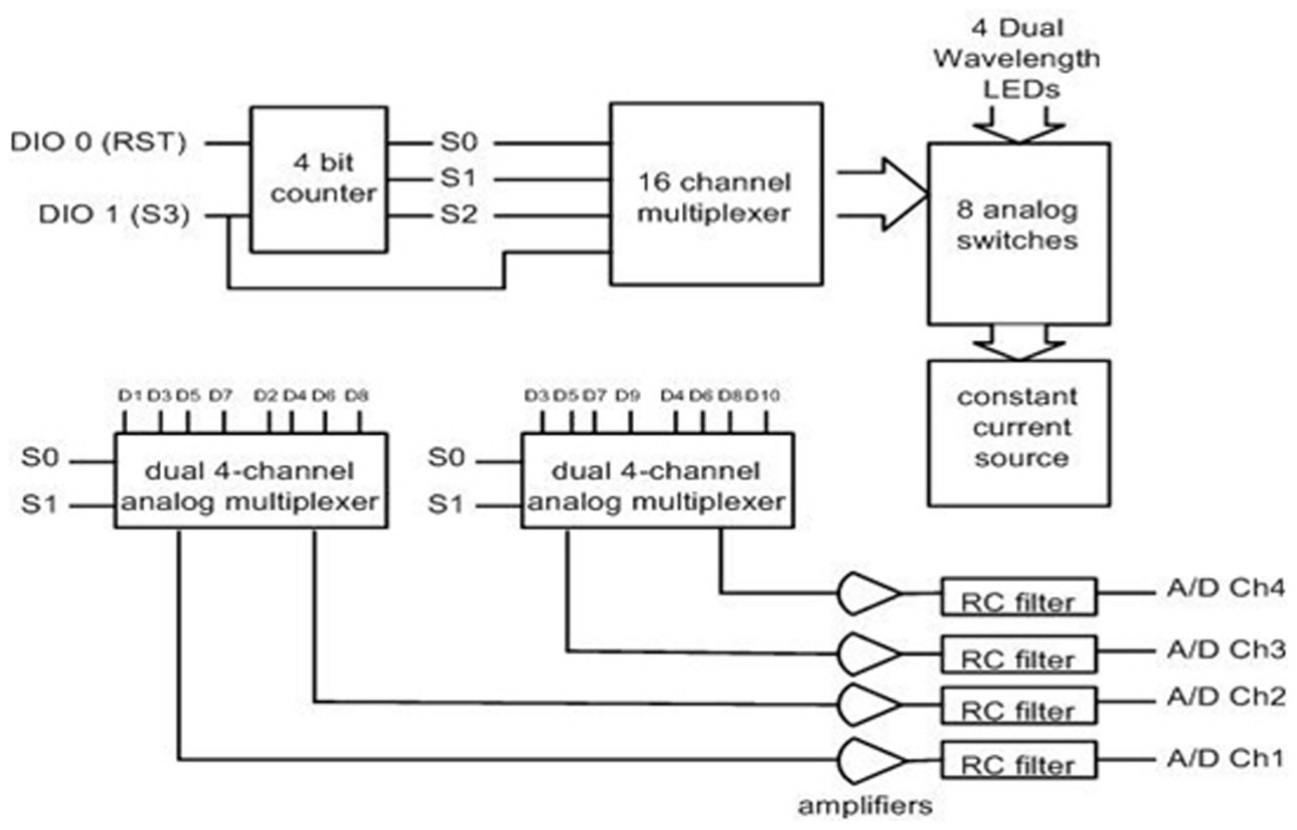
**Block diagram of the control circuit**.

The timing in the circuit is controlled only by two digital signals, DIO0 and DIO1 that feed a negative edge triggered 3 bit counter. A detailed timing diagram is presented in Figure 9, a block diagram of the circuit is given in Figure 10. One of the two digital control lines, DIO0, is the reset/initialization signal and the other one DIO1 controls the 4:16 demultiplexer and triggers state transitions as shown in Figure 10. That demultiplexer controls selection of the light source LEDs by turning on/off the single pole throw switch connecting to the current source. During a scan, one of four LEDs is lit at one wavelength at a time and the surrounding four light detectors are sampled. The operation is repeated for the second wavelength and for the other three LEDs. During a scan, background light level at each detector is measured while all LEDs are off. The outputs of the analog multiplexers which select the detector outputs are amplified, filtered and digitized. The data collected for each scan cycle is transmitted from the pocket PC to the base station computer through wireless connection for data analysis and display.

### Integrated single unit approach

**Figure 11:**
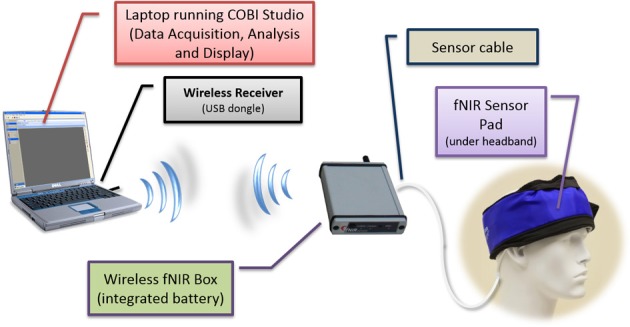
**Schematic diagram of the wireless system components**. The fNIRS box (approximately the size of a cell-phone) contains, battery, wireless transmitter, and control circuitry.

The two unit system developed earlier presented usability challenges, for example, it required participants to carry two separate hardware pieces (pocket pc and control box) and the experimenter needed to maintain and charge two separate batteries, etc. To address these challenges and further miniaturize the overall system, a unified single part wireless system was developed (Rodriguez et al., 2010; Rodriguez and Pourrezaei, 2011). The single unit wireless fNIRS system was designed to meet a series of requirements including being pocket-sized, lightweight, and compatible with operation in a hospital/clinical or other work settings where other wireless communications are taking place. Design parameters also included working with a rechargeable battery and also with a single charge, minimum of 3 h operation at maximum power consumption settings. The device was also required to interface and allow the use of the fNIRS sensor currently being used with the “wired” versions of the system developed at the optical brain imaging lab of Drexel University. Finally, the system should interface and communicate with the COBI Studio (Ayaz et al., 2011) which is a hardware integrated software platform that is used for all instruments developed at the Optical Brain Imaging Lab of Drexel University. The implemented system is depicted in Figure 11 below. It provided comparable performance to that of the wired system in terms of signal integrity and signal to noise ratio (SNR) (Rodriguez and Pourrezaei, 2011) and was used in pediatric monitoring for pain assessment of neonatal patients in intensive care units (Izzetoglu et al., 2011).

#### Implementation

**Figure 12:**
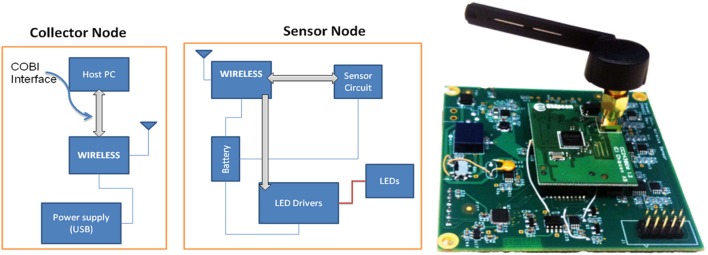
**Block diagram of the wireless fNIR controller box (left) and sensor node, circuit board implementation (right)**.

The instrumentation split into two nodes (i) a “collector node” that connects to a host computer and (ii) a “sensor node” that collects the data (Figure 12). Both nodes communicate with each other through wireless. For the wireless communication technology between these two nodes common technologies where assessed including Zigbee, Bluetooth, and Wi-Fi. Zigbee was chosen for this implementation due to its low cost, low-power requirement, and wireless mesh networking capabilities. The low power usage allows longer life with smaller batteries and the mesh networking provides high reliability and larger network range coverage.

**Figure 13:**
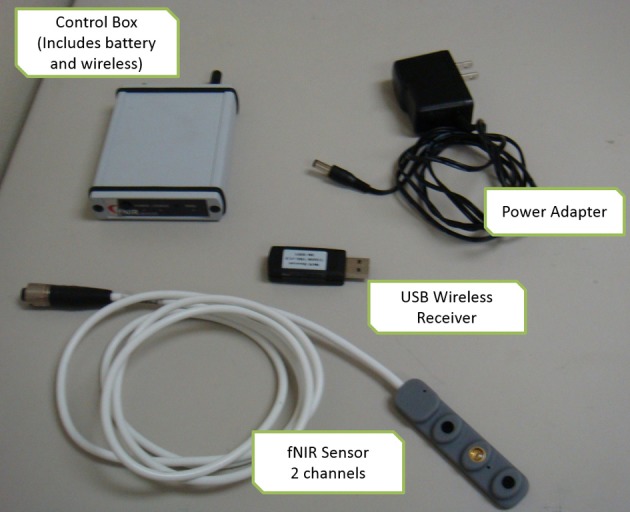
**Implemented components of the wireless system**.

The Texas Instruments CC2430 System-On-Chip (SOC) was selected for this solution; the built in 2.4 GHz IEEE 802.15.4 transceiver, 8051 enhanced core, eight channel 12-bit ADC, and 21 digital IOs were sufficient (See Figure 12). A new sensor that contains two monolithic photodiodes with built-in transimpedance amplifiers and a two-wavelength (730 and 850 nm) LED source was developed (see Figure 13). The LED driver circuit was designed to be capable of driving each wavelength separately with output currents up to 50 mA. The sensor circuit has a buffer amplifier for each of the two photodiodes, whose gain can be controlled individually by the microcontroller. The input signals from the detectors are multiplexed and then passed through anti-aliasing filter (a low pass analog filter) to remove high frequency noise before being fed to the analog-to-digital converter (ADC), which samples at a 1.5 kHz rate per channel.

An optional digital filtering of the data can be performed at the sensor node, prior to packaging and sending the data packets over the wireless network. This system can be used with various types of sensor that have different number of detectors. The “collector node” of the system communicates with a host computer through the Universal Serial Bus (USB) port utilizing a custom communication protocol that allows the COBI Studio software to control the system.

#### Evaluation of the system

Testing of the newly designed sensor was performed in terms of noise, accuracy, stability, and effectiveness of obtaining signals using solid and liquid phantoms that mimic the adult human head before measurements in humans.

***Solid phantom tests***. The SNR data calculated for using the modular fNIR sensor at a gain setting of 1 and 10 were compared for the input buffer amplifier. The SNR was higher for gain 10 and increased for higher LED current values from around 60 to 75 dB as the LED current reached 40 mA.

**Figure 14:**
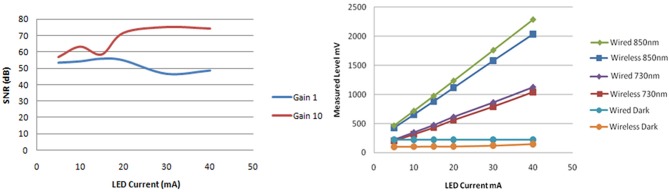
**Signal to noise ratio of one of the inputs for different LED currents and input gain settings (left)**. Comparison of wired and wireless system light intensity measurements indicated consistent response on a solid brain phantom with constant gain of 10 and LED currents ranging from 5 to 40 mA (**right**).

In Figure 14 the response of both systems are compared when the input gain setting is set to 10 and the LED current is swept from 5 up to 40 mA. It can be seen that the wireless system performs very close to the wired version: the difference between the measurements is 8% and this difference remains almost constant across the different setting configurations under which the data was collected. The gain linearity of the wireless system was also analyzed. With the LED current held constant at 20mA and the gain changed to values of 1, 10, and 20, the system responded linearly within this range.

**Figure 15:**
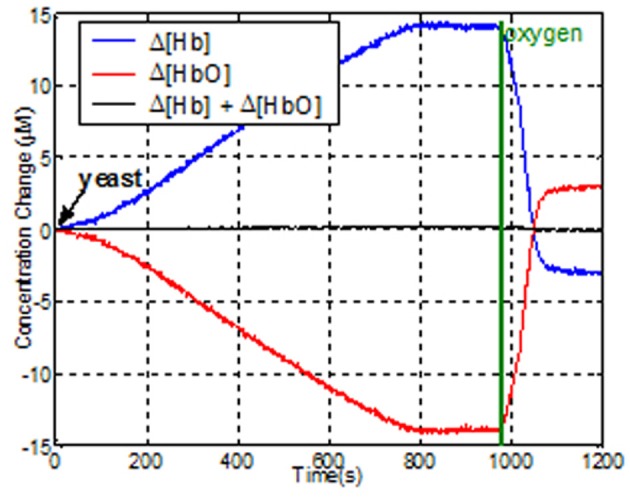
**Effects of de- and re-oxygenation during blood test on liquid phantom as measured by the wireless fNIRS system**.

***Liquid phantom tests***. To evaluate the dynamic response of the system for changes in oxygenated-hemoglobin (HbO) deoxygenated-hemoglobin (Hb) concentrations, a tissue stimulating phantom was used (Bozkurt et al., 2005; Yurtsever et al., 2006; Rodriguez and Pourrezaei, 2011). Liposyn III solution of 1% was prepared in a cylindrical transparent glass beaker from 30% Liposyn III in 1000 ml phosphate buffered saline at pH 7.4. This solution has reduced the scattering coefficient of 10 cm^−1^ at 830 nm, which is a good estimate for the human forehead. The mixture was continuously stirred with a magnetic stirring rod to keep the solution homogeneous. To simulate the blood content in tissue, around 50 μM, 22 mL of human blood was added to the beaker. The sensor pad was attached to the side of the beaker and baseline was recorded from the wireless unit using COBI Studio. Then, 4 g of baker's yeast was added to the mixture. The yeast respiration led to deoxygenation, so [HbO] decreases and [Hb] increases. Representative data is presented in Figure 15. After 13 min, the [Hb] and [HbO] reached a steady state, where oxygenation of hemoglobin and yeast respiration are at equilibrium. Then, we provided oxygen to the solution from an oxygen tank (green line in the graph) to re-oxygenate deoxyhemoglobin. As a result of oxygen bubbling inside the beaker, hemoglobin saturation exceeds the initial saturation and steady state is reached at a higher saturation level.

## Conclusions

This paper described a range of studies on human performance assessment and skill acquisition monitoring using fNIRS measures of the hemodynamic response of the prefrontal cortex and its relationship to mental workload, expertise, and performance. The results show that the effects of task load and expertise on the hemodynamic response can be reliably and sensitively assessed in a range of tasks, from standardized laboratory tasks to complex cognitive tasks representative of real work settings. With respect to the development of wireless, portable fNIRS, the results, although preliminary, corroborate previous findings and point to the potential of the fNIRS system as a wearable, portable and non-invasive sensor for future neuroergonomics studies. Moreover, miniaturization and wireless system development efforts reported here will benefit future studies that can allow participants to freely navigate in indoor or outdoor environments untethered, consistent with the MoBI approach. Although further work may be needed in specific applications, both wired and wireless fNIRS systems allow for the examination of dynamic aspects of brain function in more natural settings, and are thus suitable for reliable human performance and training assessment.

### Conflict of interest statement

fNIR Devices, LLC manufactures the optical brain imaging instrument and licensed IP and know-how from Drexel University. Hasan Ayaz, Kurtulus Izzetoglu, and Banu Onaral were involved in the technology development and thus offered a minor share in the new startup firm fNIR Devices, LLC. The authors declare that the research was conducted in the absence of any commercial or financial relationships that could be construed as a potential conflict of interest.
